# Improving cancer immunotherapy by rationally combining oncolytic virus with modulators targeting key signaling pathways

**DOI:** 10.1186/s12943-022-01664-z

**Published:** 2022-10-12

**Authors:** Zhi Zhu, A. J. Robert McGray, Weijian Jiang, Binfeng Lu, Pawel Kalinski, Zong Sheng Guo

**Affiliations:** 1 UPMC Hillman Cancer Center, Pittsburgh, PA USA; 2 Department of Immunology, University of Pittsburgh School of Medicine, Pittsburgh, PA USA; 3 Department of Surgical Oncology and General Surgery, The First Hospital of China Medical University, Shenyang, Liaoning China; 4 Department of Immunology, Roswell Park Comprehensive Cancer Center, Buffalo, NY USA

**Keywords:** Small molecule, Inhibitor, Signaling pathway, Targeted therapy, Combination regimen, Oncolytic virus, Antitumor immunity, Efficacy, Immuno-oncology

## Abstract

Oncolytic viruses (OVs) represent a new class of multi-modal immunotherapies for cancer, with OV-elicited antitumor immunity being key to their overall therapeutic efficacy. Currently, the clinical effectiveness of OV as monotherapy remains limited, and thus investigators have been exploring various combinations with other anti-cancer agents and demonstrated improved therapeutic efficacy. As cancer cells have evolved to alter key signaling pathways for enhanced cell proliferation, cancer progression and metastasis, these cellular and molecular changes offer promising targets for rational cancer therapy design. In this regard, key molecules in relevant signaling pathways for cancer cells or/and immune cells, such as EGFR-KRAS (e.g., KRAS^G12C^), PI3K-AKT-mTOR, ERK-MEK, JAK-STAT, p53, PD-1-PD-L1, and epigenetic, or immune pathways (e.g., histone deacetylases, cGAS-STING) are currently under investigation and have the potential to synergize with OV to modulate the immune milieu of the tumor microenvironment (TME), thereby improving and sustaining antitumor immunity. As many small molecule modulators of these signaling pathways have been developed and have shown strong therapeutic potential, here we review key findings related to both OV-mediated immunotherapy and the utility of small molecule modulators of signaling pathways in immuno-oncology. Then, we focus on discussion of the rationales and potential strategies for combining OV with selected modulators targeting key cellular signaling pathways in cancer or/and immune cells to modulate the TME and enhance antitumor immunity and therapeutic efficacy. Finally, we provide perspectives and viewpoints on the application of novel experimental systems and technologies that can propel this exciting branch of medicine into a bright future.

## Introduction

The mammalian immune system comprises a network of innate and adaptive cell subsets which collectively discriminate invading or arising “non-self” elements from healthy “self” components of the body to eliminate pathogens such as viruses, bacteria, parasites, or pathogenic cellular changes or features, as arise in cancer. Evolving pathogens have developed multiple sophisticated mechanisms to antagonize or even exploit host immunity to their own advantage [1, 2]. Recent developments have improved our understanding of the molecular and cellular interplay between viruses and the immune system and have led to the design of new strategies to turn viruses from stealth pathogens into finely tuned therapeutic vehicles that can promote both direct viral-mediated and secondary immune-mediated attack against cancer cells. Oncolytic viruses (OVs) represent a key example of such approaches [3].

OVs are a diverse collection of viruses being developed as versatile therapeutic platforms for treating cancer. OVs preferentially infect and replicate in cancer cells and cancer-associated stromal cells and can be engineered to express transgenes that augment their cytotoxic and immunostimulatory activities [4–9]. Importantly, in addition to direct lytic function, OVs modulate the tumor microenvironment (TME) and enhance loco-regional inflammation and immune cell-mediated tumor eradication, while also enhancing systemic cancer immunity [10, 11]. In this way, OVs have the potential to be utilized as a comprehensive and potent therapeutic platform in combination regimens [4–9, 12]. They have also undergone through clinical trials [13–15].

Small-molecule drugs targeting key cellular signaling pathways are becoming an important class of drugs for cancer therapy [16–18]. Importantly, this approach has uncovered a previously underappreciated crosstalk between tumors and the immune cells present within the TME. First, some of these modulators targeting key signaling pathways for cancer cell survival and proliferation have been shown to induce immunogenic cell death (ICD) of cancer cells, enhancing cancer immunogenicity and subsequent antitumor immunity [19–22]. Second, in addition to the expected effects of inducing death of cancer cells, targeting tumors with kinase inhibitors has been shown to reverse the immunosuppressive TME [23, 24]. Third, as both cancer cells and immune cells undergo metabolic and epigenetic reprogramming in the TME [25, 26], modulators to key enzymes involved in epigenetic and metabolic pathways have the potential to inhibit tumor cell growth and proliferation, and to restore the normal functions of immune cells [27, 28]. Based on these tumor modulating and therapeutic properties, it is logical to explore the potential of combining these agents with OV as rational approaches to cancer therapy.

We have previously published three review articles highlighting various aspects of oncolytic immunotherapy [29–31]. In these reviews, we focused on two themes. The first was on the mode of cell death induced by OVs, mostly in the form of ICD, and the significance of ICD in eliciting potent antitumor immunity and its functionality as in situ therapeutic cancer vaccines [29, 30]. The second theme was on the development of vaccinia virus for cancer vaccines and as OV [31]. In the current review, we focus on combinatorial strategies integrating OVs with modulators of key signaling pathways in cancer and/or immune cells for optimized therapeutic efficacy. As small molecule modulators have shown promising results in preclinical and clinical studies, with some gaining approval for cancer therapy [17, 18, 32, 33], combining OV with small molecule modulators as rational therapeutic combinations will be the focus of discussion.

Single agent cancer therapies often produce limited efficacy [17, 34–36], and this includes immunotherapy [35]. One of many potential causes for lack of sufficient therapeutic response is tumor heterogeneity, which leads to an incomplete response to a particular monotherapy [36]. Rational combination regimens are badly needed to overcome the heterogeneous responses currently observed to these potentially curative therapies, as drug candidates could work additively or synergistically to produce enhanced therapeutic effects [34]. Surprisingly, a recent study showed that patient-to-patient variability and independent drug action are sufficient to explain the superiority of many FDA-approved drug combinations in the absence of drug synergy or additivity [37], suggesting that it may not be necessary for drugs to have additive or synergistic effects in order demonstrate therapeutic benefit for patients. This insight represents an unusual way to design combination therapies. In summary, combination therapy such as OVs with modulators of cellular signaling pathways are highly desired, although the clear rational for combining such approaches may not be immediately obvious and may require further mechanistic insights in determining potentially impactful therapeutic strategies.

## Oncolytic viruses and immunotherapy: overview

### Basic studies of OV-mediated immunotherapy

**Fig. 1:**
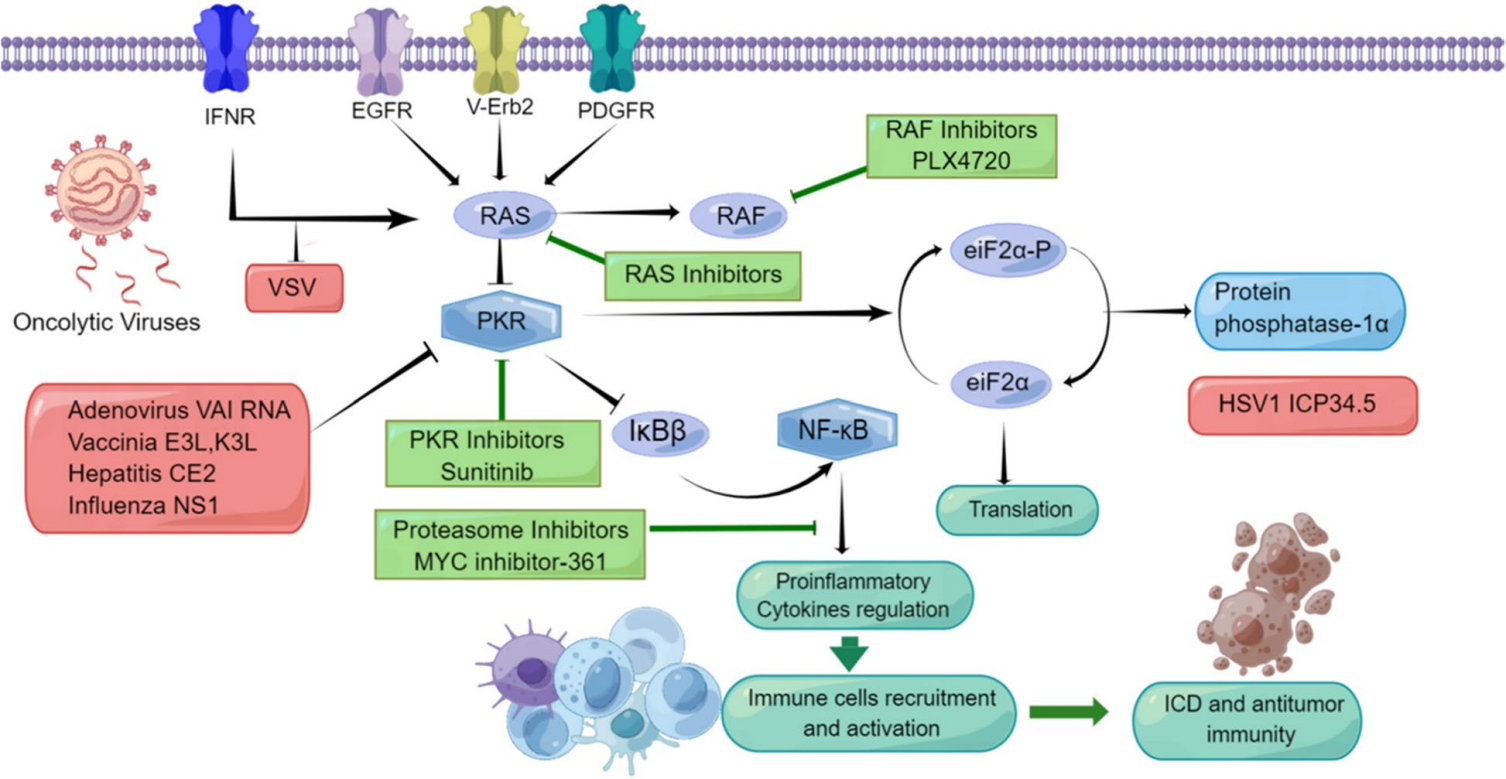
Viral proteins and small molecule compounds may inhibit signaling in synergy to promote viral replication, and improve elicited inflammation, ICD and antitumor immune responses. We use RAS, IFN and dsRNA-dependent protein kinase (PKR) pathways as an example. Most viruses replicate poorly in cells that produce active PKR. In response to viral infection, PKR activates the transcription factor NF-κB by inducing degradation of IκBβ. NF-κB activates transcription of proinflammatory genes that induce an immune response against viruses. RAS activation by EGFR, v-Erb2, or platelet-derived growth-factor receptor (PDGFR) signaling inhibits PKR activity. RAS activation therefore allows viral oncolytic activity in cancer cells. PKR activity is also activated by interferon (IFN)-α/β signaling through the IFN receptor (IFNR). Tumor cells with defects in this signaling pathway allow a higher degree of viral replication than normal cells. Several viral proteins and RNAs (adenoviral VA RNA128, VV’s E3L and K3L, the hepatitis CE2 protein and influenza virus NS1 protein) inhibit PKR. Interfering with the different steps of signaling pathways using different classes of compounds have resulted in increased viral replication and subsequent efficacy. PKR shuts off protein synthesis by phosphorylating eIF2α. The protein phosphatase-1α is activated by the protein ICP34.5, which is expressed by HSV-1. ICP34.5 dephosphorylates eIF2α to allow protein synthesis to continue. An HSV1 strain that expresses a mutant form of ICP34.5 can therefore only replicate in cells with inactive PKR. Targeting this process by means of different small molecule inhibitors increased OV spread and efficacy. Note: Viral genes are shown in red, small molecule inhibitors are shown in green, whereas cellular proteins are shown in blue

OVs preferentially infects and kills cancer cells without causing collateral harm to normal cells and tissues. As the infected cancer cells and/or tumor-associated stromal cells are destroyed by oncolysis, they release new infectious virus particles or virions to infect and destroy the remaining tumor cells/stromal cells. The tumor selectivity of OVs have been well studied in many cases [38–40]. Some viruses possess natural tumor cell tropism while others gain this property through genetic engineering [40]. A particular OV may work through one or more mechanisms. First, cellular entry via virus-specific, receptor-mediated mechanisms restricts the virus to cancer cells and cancer-associated cells. Second, rapid cell division in tumor cells with high metabolic and replicative activity may support increased viral replication compared with quiescent normal cells. Third, tumor-driver mutations specifically increase the selectivity of virus replication in tumor cells (Fig. 1). Reovirus and vaccinia virus naturally possess the ability to specially target cancer cells driven by the activated Ras pathway. Reovirus preferentially replicates in Ras-activated cells [41]. Vaccinia virus (VV) targets cancer cells that overexpress EGFR as it requires EGFR-Ras signaling to replicate [42]. The genetically engineered VV, Pexa-Vec (JX-594), targets cancer cells via multiple mechanisms, whereby virus replication is activated by EGFR/KRAS pathway signaling, cellular thymidine kinase (TK) levels, and resistance to type I interferons (IFNs) in cancer cells [42]. Finally, some OVs target cancer cells and/or tumor-associated stromal cells. Notably, multiple distinct mechanisms or OV features can underpin this process. For example, some OVs can selectively infect and replicate in stromal cells. Oncolytic vesicular stomatitis virus (VSV) infects and destroys tumor vasculature in vivo but leaves normal vasculature intact [43]. OVs expressing certain T-cell engagers simultaneously targets cancer and immunosuppressive stromal cells [44–46]. Importantly, it has been shown that stromal destruction is required for the eradication of established solid tumors by adaptive immunity involving T cells under certain conditions [47]. Therefore, those OVs that effectively target both cancer cells and stromal cells are likely to be advantageous.

OVs destroy cancer cells and/or inhibit tumor progression through four distinct mechanisms: oncolysis, vascular collapse, antitumor immunity, and expression of therapeutic transgene(s) [48]. Antitumor immune responses are potentiated through immunogenic cell death (ICD) of cancer cells and subsequent presentation of danger signals to dendritic cells (DCs), and release/presentation of cellular debris, viral and tumor antigens (including neoantigens) to the local and systemic immune cells [30, 49]. In fact, as early as 1999, Rabkin, Martuza, Toda and others have observed that an oncolytic HSV (oHSV) could function as an in-situ cancer vaccine and stimulate anti-tumor immunity [50]. In that study, OV delivery and tumor cell killing resulted in generation of CD8^+^ CTL responses against the dominant “tumor-specific” major histocompatibility complex (MHC) class I-restricted epitope (AH1) from the Gp70 antigen expressed by CT26 colon cancer cells [50]. The fact that tumor antigen-specific adaptive immunity plays a key role in OV-mediated cancer therapeutics has been verified subsequently in numerous studies. Based on these data, we and others believe that OVs function as therapeutic cancer vaccines [29, 51–53], or a specific type of immunotherapy [54].

OVs themselves can modulate the TME and turn cold tumor hot [55, 56]. The evidence comes by examining the release of DAMPs and PAMPs [30, 49, 57], the cytokine/chemokines produced [30, 49, 58], infiltration of immune cells [59, 60], induction of ICD [61, 62], activities of infiltrated immune cells and elicited antitumor immunity in tumor models [30, 49, 63, 64], among other properties after treating the tumors with OV. In order to further improve its immunostimulatory functions, several strategies have been exploited. One is simply to integrate immunostimulatory genes such as Th1-cytokines into the viral vectors [65]. This was done with GM-CSF and IL-2 in the early iterations of this approach [66, 67]. IL-10, originally considered to be a Th2 cytokine, but later shown to be a Th1 cytokine in certain environments, has been incorporated into OVs and demonstrated improved antitumor immunity and enhanced efficacy [68, 69]. IL-24, a member of the IL-10 family, has also been shown to be potent antitumor factor when expressed from an OV [70, 71]. We and others have engineered oncolytic vaccinia viruses (VVs) expressing recombinant IL-2, IL-12, IL-15, IL-21, IL-23, and IL-36γ for improved efficacy and safety in multiple tumor models [64, 72–77]. In some cases, two cytokines may complement each other and synergize to activate antitumor immunity and lead to complete tumor regression in non-immunogenic tumor models. This is the case for IL-7 and IL-12 expressed by OV [78, 79]. The second approach is to engineer co-stimulatory ligands such as ICOS ligand (ICOS-L) into OV to enhance co-stimulation of immune cells within the TME. As OV delivery often leads to upregulation of T-cell co-stimulatory receptors, with the inducible co-stimulator (ICOS) being most notable [80], this is a rational approach to further augmenting anti-tumor immunity using OV. Third, it is also possible to use the reverse approach, whereby OV can be engineered to express inhibitors or antagonists targeting co-inhibitory molecules. For example, recent studies showed that engineered OV expressing ICIs could activate anti-tumor responses [81–83]. Fourth, there are a few “don’t eat me” signals (CD24 and CD47) that that cancers seem to use to evade detection and destruction by the immune system. Thus, OVs could be engineered with an antibody against CD47 or CD24 and improved innate immunity, in addition to its known functions of oncolysis and modulation of immune cells [83, 84]. A fifth strategy is to engineer an OV with a tumor antigen, helping such armed OV elicit robust tumor antigen-specific CD8^+^ T cell responses, leading to improved antitumor therapy [85]. The sixth is to disrupt the signaling pathways that facilitate interactions between cancer cells and their environment. For example, CXCR4 is one of the key stimuli involved in signaling interactions. Studies found that targeting CXCL12/CXCR4 signaling by an OV expressing CXCR4 antagonist effectively disrupted the tumor vasculature, induced ICD of cancer cells, reversed immunosuppressive TME and improved antitumor immunity, including inhibition of cancer metastasis [86, 87]. Lastly, engineering OV to express a T-cell engager can engage naïve T cells and cancer cells, which activates these T cells to kill cancer cells, bypassing the dependence of MHC antigen on cancer cells [88–90]. In addition, OVs armed with bispecific engager targeting both T cells (CD3) and fibroblast (e.g, fibroblast activation protein) can target both cancer proper and associated stroma [89, 90].

In summary, as novel class of antitumor agents, one unique property of OVs is that they replicate selectively in cancer cells, yet express other therapeutic proteins locally to amplify its antitumor effects and modulate the TME to turn cold tumor hot. As they elicit both innate and adaptive antitumor immunity, they target tumor locally yet act systemically to inhibit/eliminate not only primary tumor, but also micro-metastases at a distance.

### Clinical studies of OV immunotherapy

Three OVs have been approved for treatment of human cancers: H101 (adenovirus, AdV), T-VEC (herpes simplex virus, HSV), and Delytact (G47∆; HSV) were approved in China (in 2005), USA (in 2015), and Japan (in 2021) [91], respectively.

As summarized in a recent review, 97 clinical trials with OVs enrolling a total of 3233 cancer patients have been completed, resulting in 119 published reports [15]. Among these studies, objective clinical responses were reported in only 9% of patients and disease control was achieved in only 21% of patients, suggesting a clear need to enhance therapeutic responses to OVs. Three GM-CSF-armed OVs highlighted clinical studies and progression of the field. They are genetically engineered from HSV-1 (T-VEC), human adenovirus (CG0070), or vaccinia virus (Pexa-Vec), respectively. For the approved T-VEC, local and distant antitumor immunity was induced by intralesional vaccination with the virus in patients with stage IIIc and IV melanoma [92, 93]. This antitumor immunity was associated with overall objective clinical response that led to approval of T-VEC by the FDA for patients with advanced melanoma [94]. A recent study in melanoma patients of stage IIIB-IVM1a with injectable, unresectable metastatic lesions demonstrated that treatment of a “real-life” cohort of patients with T-VEC resulted in high overall response rate (64%) and a large fraction of durable complete responses (43%) [95]. However, as observed in the randomized, double-blind phase III trial (NCT02263508), combining T-VEC with pembrolizumab did not lead to a survival benefit compared to pembrolizumab alone for patients with advanced melanoma [96]. The second OV, CG0070, has undergone phase II testing in patients with BCG-unresponsive non-muscle-invasive bladder cancer. In this patient cohort, intravesical CG0070 yielded an overall 47% complete response (CR) rate at 6 months for all patients and 50% for patients with carcinoma-in-situ, with an acceptable level of toxicity [97]. This OV is currently being evaluated in the phase III BOND-003 trial (NCT04452591), and in phase II trial in combination with pembrolizumab. The preliminary report of the phase II trial showed a CR rate of 88% (14/16) at the 3 month assessment interim timepoint [98]. Finally, Pexa-Vec demonstrated oncolytic and immune-mediated mechanisms of action, tumor responses and dose-related survival in individuals with hepatocellular carcinoma (HCC) in a phase 2 trial [99]. In the phase III PHOCUS trial (NCT02562755), unfortunately Pexa-Vec/Nexavar combination therapy failed to meet the intended criteria (mainly improved clinical benefit when compared to Nexavar alone). However, 10 additional clinical studies in phase I/II are evaluating Pexa-Vec in other solid cancer indications and may reveal additional therapeutic opportunities for this OV. For example, a phase I/II study of Pexa-Vec in combination with immune checkpoint inhibitor (ICI) in refractory colorectal cancer (NCT03206073) may demonstrate that this combination is efficacious [100]. In summary, OV-elicited antitumor immunity contributes significantly to, or is essential for the overall therapeutic efficacy mediated by an OV. However, other mechanisms of action, such as cytotoxicity and anti-angiogenesis, also contribute to the overall therapeutic effects and represented additional opportunities to enhance the effectiveness of OVs. As monotherapy, OVs exert limited efficacy and thus rational combination strategies are desperately needed to further improve the efficacy of this novel class of immunotherapy.

In regard to OV toxicity, multiple studies have investigated this potential therapeutic challenge using animal models. For example, Lun et al. have studied the toxicity of Pexa-Vec (JX-594) [101]. A supratherapeutic dose of JX-594 demonstrated GM-CSF-dependent inflammation and necrosis in non-tumor-bearing rodents. In another study, Tang et al. found that some brain tumor-bearing mice died soon after treatment with vvDD-IL15-Rα, and close examination revealed that viral infection of ependymal cells, subventricular cells, and meninges was widespread, leading to death [102]. VSV exhibits natural neurotropism, but genetic engineering can abrogate neurotoxicity [103]. So far clinical trials with OVs have not shown significant toxicity or safety issues. Influenza-like symptoms (such as chills and fever) have been noted for both local and systemic administration of OVs but are mild [104, 105].

### Biomarkers for OV immunotherapy

As only a fraction of patients treated with OV go on to derive treatment benefit, it stands to reason that identifying clinical biomarkers that can successfully predict patients who will respond favorably will improve outcomes to OV therapy [106]. These may include both predictive and response biomarkers, and they may guide OV therapy [106].

Cancer cells possess multiple signaling or cellular hallmarks [107] that can be exploited for OV infection, replication and oncolysis (Fig. 1). It has been shown that defects in interferon pathways potentiate the sensitivity of cancer cells to various OVs, including VSV [108]. Many OVs depend on oncogenic signaling pathway that are constitutively activated in cancer cells for their selective viral replication and oncolysis. For example, a number OVs depend on activated Ras pathway for their replication, including reovirus [109], influenza A virus delINS1 [110], poxvirus Pexa-Vec [42], coxsackievirus Type B3 [111], and alphavirus M1 [112]. For alphavirus M1, there have been four biomarkers identified thus far that correspond to increased OV activity: zinc-finger antiviral protein (ZAP), inositol-requiring kinase 1α, Ras homolog family member Q, and mutated and activated KRAS [113].

Until now, a limited number of response biomarkers for OV-mediated therapy have been identified [6]. The first one may be immunoglobulin-like transcript 2 (ILT2) for oncolytic VV [114]. The Kaufman lab observed an inverse association between ILT2 expression in the tumor and clinical response. They further identified ILT2 as a marker of regulatory CD4^+^ and suppressor CD8^+^ T cell responses, and ILT2 down-regulation was predictive of therapeutic responses in patients treated with oncolytic VV-mediated immunotherapy. Serum HMGB1 may be a predictive and prognostic biomarker for immunotherapy with oncolytic adenovirus [115]. Additionally, Nguyen and colleagues showed that defects in IFN-JAK-STAT pathway as response biomarkers to virotherapy mediated by oncolytic VSV and HSV-1 [116]. Another recent study identified the receptor of oncolytic alphavirus M1 as a therapeutic predictor for multiple solid tumors [117]. In summary, we believe that some of these biomarkers are tumor type-specific and/or OV-specific. As summarized correctly by Kaufman [106], each OV is unique and contains a different set of viral genes, other genetic components, and arming transgenes. These genetic factors and/or strategies to modify the OV may influence their biologic interactions in different tumors, and impact different gene expression status within infected cells, including cell death pathways. As such, we need to define how changes in specific innate sensing and antiviral machinery elements influence the ability of specific OVs to infect and replicate in individual tumor cells and how these changes impact antiviral and antitumor immune responses by the host. In addition to validating these identified biomarkers, uncovering additional biomarkers that can reliably predict therapeutic response will be of tremendous value.

In the following section, we will discuss biologically relevant and targetable signaling pathways and the development status of the current and emerging inhibitors/modulators. We then focus on rational combinations, including those that produce mixed responses when combined with OV, where OVs combined with inhibitors/activators of signal transduction pathways have been evaluated in preclinical studies or advanced to clinical testing.

## Signaling pathways, modulators and combinations with OVs for improved therapy

Cancer development is driven by genetic and epigenetic alterations that allow cells to proliferate abnormally and escape mechanisms that normally control their survival and migration. Many of these alterations can be attributed to signaling pathways that control cell growth and signaling networks that fuel cancer progression [107, 118]. In addition to the well-known original hallmarks of cancer [107], it is intriguing to note that cancer cells display cancer-associated metabolic changes, or hallmarks of cancer metabolism [119, 120]. Importantly, it is not only cancer cells, but also immune cells in the TME that undergo metabolic reprogramming some of which may lead to immune tolerance [121]. Thus, these key-signaling molecules including metabolic enzymes in cancer cells, immune cells, and likely stromal cells are all potential therapeutic targets.

Many investigators have focused on the discovery and exploration of small molecules to modulate key signaling pathways as cancer treatments. Many small molecules can effectively modulate immune responses and the immunosuppressive TME [122]. Overall, data thus far has demonstrated a series of successful targeting, future therapeutic opportunities, as well as potential challenges [32, 33]. Some investigators consider the development of these small molecule drugs to be the next generation of immunotherapy for cancer [123]. Readers are referred to recent reviews related to small molecule modulators of signaling pathways and their expanding role in immuno-oncology [18, 28, 122, 123].

**Fig. 2:**
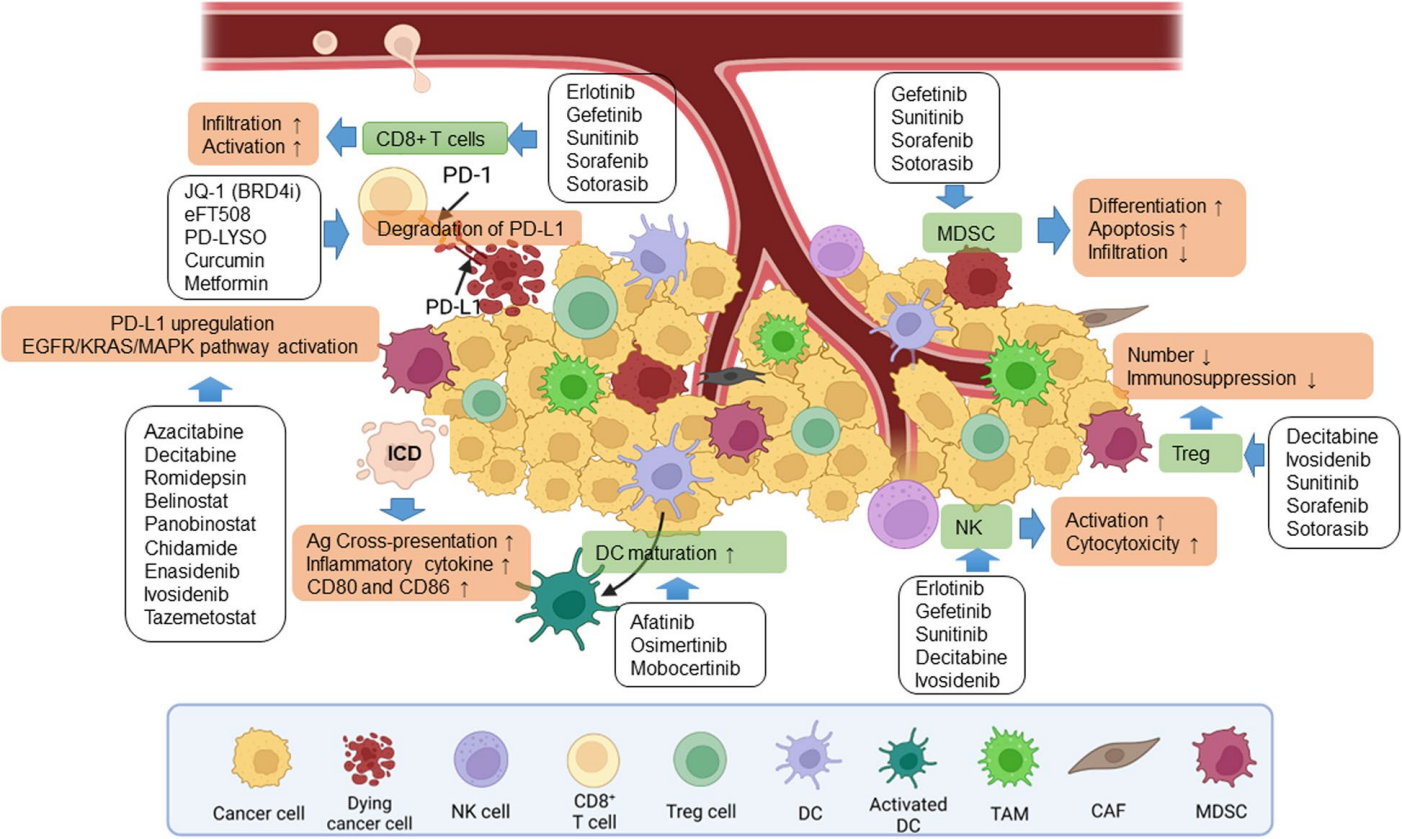
The Effects of small-molecule drugs on the TME. Targeted therapies have been shown to alter the TME in multiple ways, either directly or indirectly. Some agents could reverse the immunosuppressive environment by inhibiting the infiltration and function of MDSCs and Tregs. Numerous therapies increase the expression and presentation of tumor antigens, increasing cross-priming of DCs for enhanced T cell activation. Targeted therapies also can increase the expression of NKG2D ligands on NK cells, which serve as co-stimulatory molecules for CTL,as well as activators of NK cells, which also increase the NK cytotoxicity

In this section, we will explore key signaling pathways as well as how these pathways could be explored as targets for cancer therapy (Fig. 2). As the body of work in this area is extensive and includes a large list of both targetable pathways and candidate therapeutics, it is not possible to comprehensively review all potential approaches currently under development. As such, we focus specifically on those small molecules targeting signaling pathways that may involve antitumor immunity. Then in the second part of each sub-section, we discuss the combination strategies and studies of OV with modulators of that signaling pathway and relevant findings in preclinical studies.

### The rationale for combining OVs with small molecule modulators

**Fig. 3:**
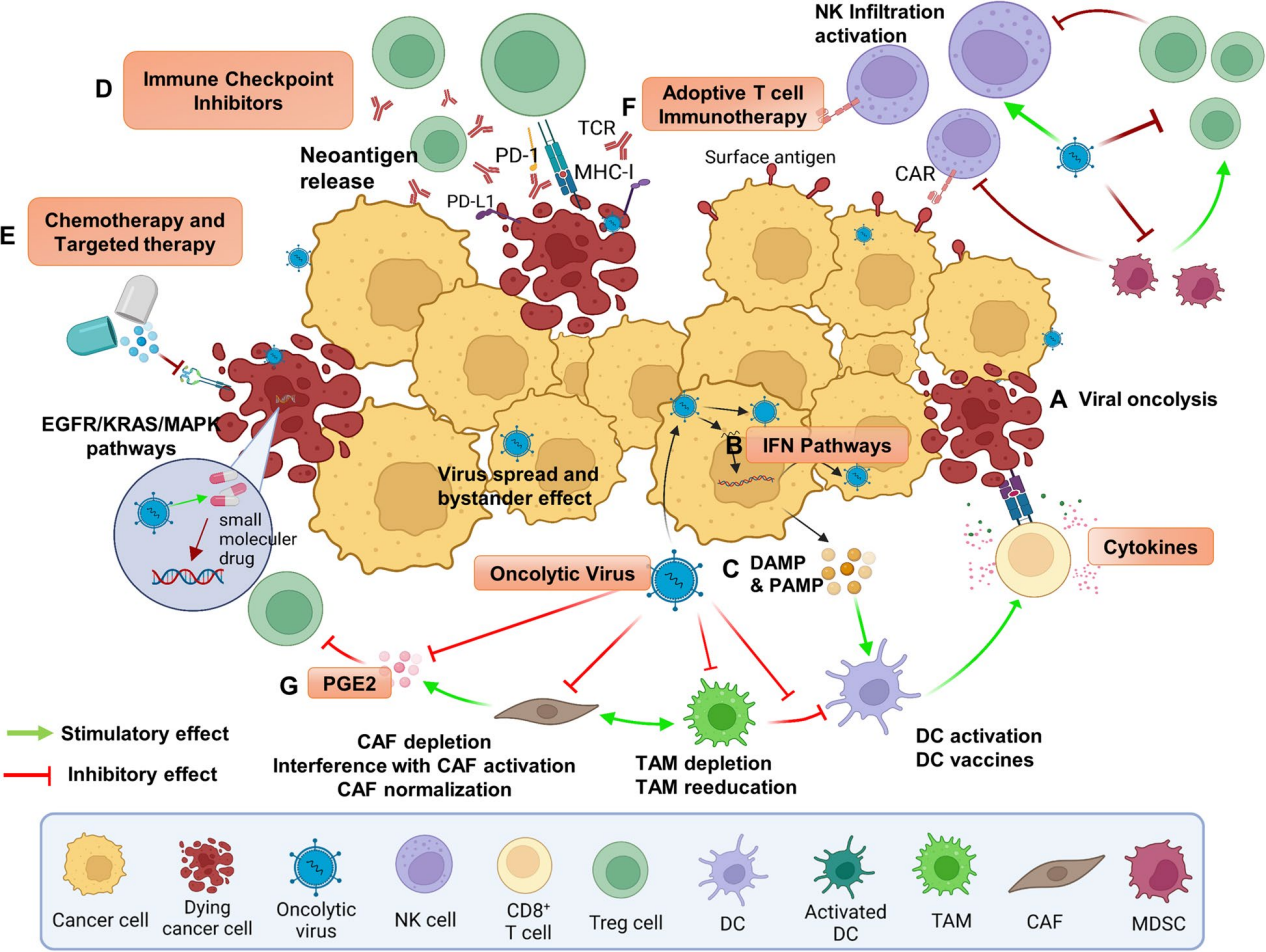
Rationale for the combination of OV with immuno-and targeted therapies in the TME for cancer. The TME is composed of diverse cell types, secreted factors, and extracellular matrix that provide targets for combination of OV therapies. We could arbitrarily divide these targets and mechanisms of action into steps A-G. A. OV replicate selectively in tumor and have the capacity for direct oncolysis. B. OV-mediated increases in the release of DAMPs, PAMPs and cytokines promote the accumulation of CTLs at tumor beds and retention of their killing capability. C. OV induce IFN pathways followed by elicitation of immune responses, thus mediating a broader range of long-lasting antitumor effects. D. OV infection leads to increased expression of immune checkpoint molecules such as PD-L1 and CTLA-4 from cancer and stromal cells, that sensitizes infected tumors to ICI. E. Cytotoxic chemotherapy destroys tumor cells by induction of cell death, often via ICD, or targeted therapies interrupt aberrant signaling pathways and potentially death of cancer cells. This may induce weak or moderate immune responses against tumor. F. Relevant cells such as TAMs, DCs, CAFs, and MDSCs secrete ECM components, growth factors, and cytokines, which can contribute to the regulation of tumor progression and therapeutic response in unique ways, such as CAFs suppress T and NK cells via cytokines and growth factors including PGE2 and TGF-β. Some OVs are designed to target not only cancer cells, but also stromal cells (e.g., CAFs). G. OV shape the TME for immunotherapy by shifting the tumor status from ‘cold’ to ‘hot’, thus, improving immune cell recruitment and effector function

Many cell types and molecules play roles in shaping the immunosuppressive TME [124]. Those cell types and molecules discussed earlier may have fundamental roles in the TME. As stated earlier, OVs themselves can modulate the TME and turn cold tumor hot [55, 56]. However, the strength of immunogenic ‘hot’ property and antitumor immunity elicited by OVs may not be strong enough to eliminate the primary tumor and secondary metastasis, and thus combination with other antitumor agents deem necessary in most cases, especially for reversing the immunosuppressive nature of the TME. Many small molecules can effectively modulate immune responses and the immunosuppressive nature of the TME [122, 125, 126]. Some investigators consider the development of these small molecules to be the next generation of immunotherapy for cancer [18, 123]. Due to their unique mechanisms of action and anti-tumor properties, these agents may act in synergy with OVs, leading to enhanced antitumor immunity and therapeutic efficacy (Figs. 2 and 3).

#### EGFR/KRAS/MAPK signaling pathway

The epidermal growth factor receptor (EGFR) family is among the most investigated receptor tyrosine kinase (RTK) groups owing to its general role in signal transduction and oncogenesis. A large fraction of human cancers displays elevated EGFR and kinase activity through overexpression and/or mutations [127]. EGFR is one of the most frequently altered oncogenes in solid cancers. Therefore, efforts have been taken to develop small-molecule inhibitors as well as large molecules or biologics (such as antibodies) to inhibit this survival and activation signaling pathway for cancer cells. For a comprehensive review of small molecule inhibitors targeting the EGFR/ErbB family of RTKs, please refer to a recently published review article [128]. Regarding toxicities related to treatment with TKIs, it is important to note that treatment was almost uniformly associated with considerable toxicities [129], with the most frequent AEs including hand–foot syndrome, diarrhea, and nausea/vomiting. Therefore, careful consideration needs to be taken when considering combination therapy using these TKIs and other inhibitors.

Since 2004, several small molecules functioning as EGFR tyrosine kinase inhibitors (TKIs) have been approved by the FDA and other authorities as cancer therapies, particularly for the treatment of non-small cell lung cancer (NSCLC) (Table 1). For example, Erlotinib was initially approved to treat NSCLC in 2004, then approved to treat pancreatic cancer in 2005.

**Table 1:** The FDA-approved inhibitors for EGFR, other RTKs and KRAS for cancer treatments

*Name*	*Target*	*Disease setting*	*Approval date*
Erlotinib (Tarceva)	EGFR-TK	Locally advanced or metastatic NSCLC; Pancreatic cancer	2004 (NSCLC)
			2005 (Pan)
Gefetinib (Iressa)	EGFR-TK	NSCLC with EGFR exon 19 deletions or exon 21 L858R mutation	2015
Afatinib (Gilotrif®)	EGFR-TK	NSCLC with non-resistant EGFR	2018
Osimertinib (Tagrissob®)	EGFR-TK	Metastatic NSCLC with EGFR exon 19 deletions or exon 21 L858R mutation	2018
Mobocertinib (Exkivity™)	EGFR-TK (orally)	NSCLC with EGFR exon 20 insertions	2021
Sunitinib (Sutent)	RTK	Gastrointestinal stromal tumor (GST), renal cell carcinoma (RCC), pancreatic cancer (Panc)	2006 (GST; RCC)
			2011 (Pan)
			2017 (Recurrent RCC)
Sorafenib (Nexavar)	RTK	Kidney, liver, and thyroid cancers	2005 (RCC)
			2007 (HCC)
			2013 (Thyroid)
Sotorasib (Lumakras™)	KRAS^G12C^	NSCLC with KRAS^G12C^ mutation	2021

Two small molecule drugs, sunitinib and sorafenib, inhibits cellular signaling by targeting multiple RTKs (Table 1). Sorafenib is an inhibitor with activity against many protein kinases, including VEGFR, PDGFR and RAF kinases [130]. Both sunitinib and sorafenib were approved for treating patients with advanced renal cell carcinoma (RCC). Sorafenib has also been approved for advanced primary liver cancer, FLT3-ITD positive AML and radioactive iodine resistant advanced thyroid carcinoma.

KRAS was considered to be undruggable until recently. Rigosertib (RGS) is a non-ATP-competitive small molecule RAS mimetic. It has the potential to block RAS-RAF-MEK-ERK and PI3K-AKT-mTOR signaling pathways and to interfere with CRAF interaction with polo-like kinase 1 (PLK1). Mechanistically, the RAS-mimetic disrupts RAS association with effector proteins to block signaling. In one study, the authors showed that RGS inhibits tumor growth in models of implanted colorectal and lung cancer, and it blocks tumor growth of a transgenic model of KRAS^G12D^-induced pancreatic cancer in mice [131]. Yan et al. have recently shown that RGS treatment led to induction of CD40 as a result of RAS/RAF/PI3K pathway disruption, followed with cancer cell death through ICD, and augmented cancer response to checkpoint blockade [132].

The progress towards developing small molecule inhibitors for mutant KRAS, particularly KRAS^G12C^, has been even more striking [133]. Sotorasib (AMG510; Lumakras™) and Adagrasib (MRTX849) have demonstrated potent clinical efficacy and high selectivity in human patients with KRAS^G12C^-driven cancers. This has been an unprecedented breakthrough. Sotorasib has been rapidly progressed from preclinical to clinical studies, to accelerated approval by FDA. Scientists at Amgen Inc. discovered and assessed the activity of this covalent inhibitor of KRAS^G12C^ [134]. In immune-competent mice, Sotorasib treatment initiated the conversion to a pro-inflammatory TME and generated durable cures alone or in combination with ICIs. In a preclinical study, Sotorasib treatment led to the regression of KRAS^G12C^ tumors and improved the efficacy of chemotherapy and targeted agents in mice [135]. A phase 1 trial of sotorasib in 129 human patients with advanced solid tumors harboring the KRAS^G12C^ mutation was published in 2020 [136]. The study showed 32% objective response and 88% disease control in the subgroup of patients with NSCLC. In a phase II trial, sotorasib therapy resulted in durable clinical benefit without new safety signals in patients with previously treated KRAS^G12C^-mutated NSCLC [137]. In 2021, the FDA approved Sotorasib for use in patients with KRAS^G12C^-mutated NSCLC (Table 1). Adagrasib has also proceeded through phase I-II clinical trials and showed clinical efficacy without new safety signals in patients with advanced KRAS^G12C^ solid tumors [138, 139]. Based on these promising clinical outcomes, two phase III trials have been planned [138].

#### Combination of OVs with inhibitor of EGFR/KRAS/MAPK signaling pathway

Investigators have tested OV combined with a small molecule inhibitor of one of the RTKs as rational combination therapy. Malignant peripheral nerve sheath tumors (MPNSTs), driven in part by hyperactive Ras and EGFR signaling, are often incurable. Cripe and his team developed a xenograft model of human MPNST and evaluated the combined antitumor effects of oHSV and the EGFR inhibitor, erlotinib. oHSV injection exhibited more dramatic antitumor activity than erlotinib. Combination therapies showed a trend toward an increased antiproliferative effect [140]. In another study with a peritoneally disseminated model of human xenograft pancreatic cancer, Canerpaturev (C-REV) combined with erlotinib had no beneficial effect on survival. However, in the subcutaneous tumor model, this combination resulted in the inhibition of tumor growth to a greater extent than using either agent on its own [141]. At present, the combination of an OV with mAb against EGFR have generated greatly improved therapeutic results in multiple studies [142, 143]. In our opinion, more definitive studies will be needed using the currently greatly improved small molecule inhibitors [128], to determine the future potential of this approach in the context of small molecule inhibitors.

Combining inhibitors of other TKRs with OVs has also been explored. For example, VEGFR TKI axitinib and oHSV have been evaluated in vitro and then assessed in two orthotopic glioblastoma (GBM) models derived from GBM stem-like cells [144]. The results showed that systemic TKI (Axitinib) beneficially combined with G47Δ-mIL12 to enhance antitumor efficacy. In another study, the authors showed that OV and SCF-1R inhibition (with PLX3397) reprogrammed the TME, enhancing the infiltration of CD8^+^ T cells and improving the impact of anti-PD-1 immunotherapy [145].

As mentioned, sunitinib is an inhibitor for multiple RTKs and its potential for use in combination with OVs has been explored by two groups. In the first study, sunitinib improved VSV-mediated virotherapy through inhibition of antiviral innate immunity [146]. In the second study, the authors explored its use together with an oncolytic VV [147]. The oncolytic VV mpJX-594 targets tumor blood vessels, spreads secondarily to tumor cells, and produces widespread CD8^+^ T-cell-dependent tumor cell killing in primary tumors and metastases. Importantly, these effects can be amplified by coadministration of sunitinib. Importantly, this study revealed multiple unrecognized features of the antitumor properties of oncolytic VV, all of which can be amplified by the multitargeted kinase inhibitor sunitinib [147].

Many cancers are driven by oncoproteins and some OVs selectively replicate in and destroy cancer cells overexpressing oncoproteins, suggesting a potentially exploitable therapeutic opportunity. It has been known for over 2 decades that human reovirus requires an activated Ras signaling pathway for infection of cultured cells, creating a clear opportunity to treat Ras activated cancers with this OV [148]. Further studies indicated that Ras-transformation affects multiple steps of the virus life cycle, including viral uncoating and disassembly, releasing PKR-induced translational inhibition, production of viral progeny, release of progeny, and viral spread following reovirus-induced cancer cell death occurring through necrotic, apoptotic, and autophagic pathways [149]. Another study suggested that reovirus induced cell death is immunogenic [150]. In this context, it is exciting to note that tumors driven by RAS signaling display a natural vulnerability to oncolytic alphavirus M1 [112]. Inhibition of the RAS/RAF/MEK signaling axis suppresses M1 infection and the subsequent cytopathic effects [112]. As such it would not be logical to combine these OVs with RAS inhibitors, highlighting that a clear understanding of the mechanistic interplay between OV and small molecule inhibitors is necessary in designing effective and potentially synergistic combination therapies.

Melanomas often have mutations in BRAF and RAS, and investigators have explored the use of BRAF/MEK inhibitors to treat melanoma in preclinical and clinical studies [151]. In one study, Roulstone et al. explored the combination of an oncolytic reovirus (TR3D) with BRAF- and MEK-targeted inhibitors in vitro and in tumor models [152]. Combined this OV with a BRAF inhibitor (PLX4720) led to significantly increased antitumor activity in BRAF mutant tumors in both immune-deficient and immune-competent models.

T-VEC has been approved for advanced melanoma, yet therapeutic responses to this OV are limited. Trametinib is a MEK inhibitor also approved for treatment of melanoma and in a study by Bommareddy et al., the combination of T-VEC and trametinib resulted in enhanced melanoma cell death in vitro [153]. The combination treatment resulted in delayed tumor growth and improved survival in tumor models. Mechanistically, regression of treated tumors was dependent on activated CD8^+^ T cells and Batf3^+^ DCs. Interestingly, authors also observed antigen spreading and induction of an inflammatory gene signature, including PD-L1. Adding anti-PD-1 antibody to T-VEC + MEK inhibition further augmented responses through enhanced tumor antigen-specific T cell responses. Interestingly, MEK inhibitor (trametinib) also enhances oncolytic VV viral replication in doxorubicin-resistant ovarian cancer cells and the combined approach attenuated A2780-R ovarian cancer growth [154]. The preclinical studies by Bommareddy et al. [153] and others, strongly support clinical evaluation of this triple combination as a rational approach in melanoma and other cancers [155].

#### PI3K/AKT/mTOR pathway

The PI3K/Akt/mTOR pathway regulates cell proliferation, growth, cell size, metabolism, and motility [156]. In many cancers such as breast, lung ovarian and prostate, this pathway is often activated aberrantly [156–158]. The enhanced activity of this crucial intracellular signaling pathway is often associated with tumor progression, and cancer’s resistance to therapies, and thus targeting this signaling pathway is a rational approach for cancer therapy [159]. There are 4 different isoforms of PI3K: alpha, beta, delta, and gamma. Small molecule inhibitors against a specific isoform of the enzyme, or as pan inhibitor, have been developed and evaluated in preclinical models with some progressing to advanced phase clinical trials [156–158].

Macrophage PI3Kγ drives progression of pancreatic ductal adenocarcinoma, and possibly other cancers [160]. Importantly, while the Syk-PI3Kγ axis in macrophages has been reported to inhibit antitumor immunity, SRX3207, a novel dual Syk-PI3K inhibitor, has been shown to block these inhibitory effects, thereby relieving tumor immunosuppression [161]. In another study, authors’ demonstrated that targeting PI3K-γ with a selective inhibitor (IPI-549) reshapes the immune milieu of the TME and promote CTL-mediated tumor regression without targeting cancer cells directly [162]. IPI-549 can inhibit PI3Kγ in MDSCs, resulting in downregulation of arginase 1 (Arg-1) and ROS to promote MDSCs apoptosis and reduce their immunosuppressive activity to CD8^+^ T cells [163]. This inhibitor is currently being evaluated in human cancer patients in multiple clinical trials.

Three different PI3K inhibitors have been approved by the FDA for the treatment of follicular lymphoma [164]. Following a successfully phase III clinical study [165], the FDA approved the first PI3K inhibitor, Piqray (alpelisib), for breast cancer patients with advanced disease and where their tumors have the PIK3CA mutation and are hormone receptor (HR) positive and HER2 negative in 2019. However, the overall landscape and therapeutic potential, as summarized by Mishra et al., is that “very few PI3K inhibitors are approved by the FDA as PI3K inhibitors suffer from many adverse effects, and poor solubility and permeability” [158].

AKT, as a key component of the PI3K/AKT/mTOR signaling pathway, exerts a pivotal role in cell growth, proliferation, survival, and metabolism [166, 167]. Small molecule inhibitors for AKT have been synthesized and evaluated in both preclinical and clinical trials [167, 168]. Capivasertib is a potent selective oral agent and inhibits all three isoforms of the AKT kinase. In the phase II FAKTION trial with postmenopausal women who have inoperable breast cancers that are aromatase inhibitor–resistant and estrogen receptor (ER)-positive/HER2-negative, Jones et al. found that the addition of capivasertib to endocrine therapy with fulvestrant prolonged progression-free survival in these patients [169]. At this time, only a few AKT inhibitors have been approved for cancer treatment [168].

Several mTOR inhibitors have been approved to treat human cancer [170]. The FDA-approved mTOR inhibitors include Sirolimus for treating patients with lymphangioleiomyomatosis with gene mutations of the tuberous sclerosis complex 2 gene in RCC, and Everolimus for RCC, pancreatic, and breast cancers. Currently, additional mTOR inhibitors are being evaluated in clinical trials. In general, it appears that mTOR inhibitors have mixed efficacy in patients across tumor indications and among patients with the same type of cancer. While mTOR inhibition alone has clear efficacy in some types of cancer, preclinical studies demonstrate strong rationale for combining mTOR inhibitors with other drugs, including OVs. While therapeutic efficacy has been demonstrated, small molecule inhibitors for the PI3K/AKT/mTOR pathway can exert certain toxicities, and the mechanisms behind these effects have been described [171].

#### Combination of OVs with inhibitors of PI3K/AKT/mTOR pathway

GBM is a lethal primary brain cancer with a median survival of less than 2 years. Rabkin, Martuza, and their teams showed that oHSVs could synergize with PI3K/AKT pathway inhibitors to target glioblastoma (GBM) stem cells [172], and prostate cancer stem-like cells [173]. Similarly, these small molecule inhibitors have also been incorporated into therapeutic regimens using other OVs, such as adenovirus [174], and Newcastle disease virus (NDV) [175]. However, not all the combinations would generate a synergy. For example, temozolomide has been used as a standard care for GBM, however it can only extend overall survival to a few months. Thus, investigators have tried to combine it with other antitumor agents for improved efficacy. A recent study found that temozolomide induces activation of Wnt/β-catenin signaling in glioma cells via PI3K/Akt pathway [176]. Yet temozolomide antagonize oncolytic immunotherapy in GBM using G47Δ-IL12 [177].

ICI is ineffective in GBM with PTEN deficiency [178]. Xing et al. have explored the impact of PTEN deficiency on OV therapy. They found that OV and PI3K inhibition work synergistically on the TME and restore immune checkpoint therapy response in PTEN-deficient GBM [179].

Wang and his team demonstrated improved systemic delivery of an oncolytic VV by using an inhibitor of PI3Kδ [180]. Transient inhibition of PI3Kδ with PI3Kδ-selective inhibitor IC87114 or the clinically approved idelalisib, enhanced the delivery and therapeutic effects of intravenously delivered oncolytic VV. This occurred by inhibiting attachment of the virus to, but not internalization by, systemic macrophages through perturbation of signaling pathways involving RhoA/ROCK, AKT, and Rac. They also applied this approach to increase the potential for intertumoral and intratumoral spread of oncolytic VV, effectively treating pancreatic cancer [181].

Rapamycin, or Sirolimus, is a macrolide compound and has immunosuppressant functions in humans and is especially useful in preventing the rejection of kidney transplants. The mammalian target of rapamycin, or mTOR, play a central role in Akt-mediated cell proliferation, differentiation, maturation and survival [182].

In 2005, Iggo and the team found that RAD001 (Everolimus), an mTOR inhibitor, improves the efficacy of oncolytic adenoviruses that target colon cancer [183]. They believed that RAD001 has three useful properties: inhibiting tumor cell growth directly, blocking angiogenesis, and suppressing the immune response. However, how RAD001 enhanced treatment efficacy was not completely understood. In 2007, McFadden, Forsyth and colleagues found that rapamycin increased myxoma virus tropism for human cancer cells and thus enhanced oncolytic virotherapy [184, 185]. Later, this combination was also applied to VV [186], HSV [187, 188], vesicular stomatitis virus (VSVΔM51) [189], and AdV [190]. As the PI3K/AKT/mTOR signaling pathway is multifunctional, it is not surprising that rapamycin could promote oncolytic virotherapy through multiple mechanisms. First, in the case of HSV and myxoma viruses, rapamycin functions to enhance the permissiveness of cancer cells to OV by reconfiguring the internal cell signaling environment to one that is optimal for productive virus replication [184, 187]. Second, rapamycin increases viral replication by impairing mTORC1-dependent type I IFN production and a reduction of intra-tumoral infiltration of macrophages [189, 191]. Third, active-site dual mTORC1 and mTORC2 inhibitors (but not rapamycin) augment HSV1-dICP0 infection in cancer cells via the eIF4E/4E-BP axis [188].

#### JAK-STAT3 pathway

Many cytokines function as crucial drivers of cancer as well as autoimmune conditions. They bind to receptors and trigger signaling cascades through Janus kinase (JAK) and signal transducer and activator of transcription (STAT) pathways. IL-6/JAK/STAT3 signaling acts to drive cancer cell proliferation, survival, invasiveness, and metastasis, while strongly suppressing antitumor immunity [192]. Thus, targeting JAKs and STATs could be an efficacious strategy [192, 193].

Currently, only four JAK inhibitors (ruxolitinib, tofacitinib, baricitinib and fedratinib) are approved for the treatment of myeloproliferative neoplasms and other disorders [194]. Many innovative studies and approaches to developing small molecule inhibitors/activators of this signaling pathways have been performed. In 2008, Xiong et al. demonstrated that inhibition of JAK1/2 signaling with AG490 (and inhibition of STAT3 as well) induced apoptosis, cell cycle arrest, and reduced tumor cell invasion in colorectal cancer cells [195]. However, despite some promising preclinical results, to date no clinical studies have shown efficacy in solid tumors treated with JAK inhibitors.

Therapeutically exploiting STAT3 activity in cancer appears to be more promising therapeutic approach [196, 197]. In 2012, Zhang et al. reported on an orally bioavailable small-molecule inhibitor of transcription factor STAT3, BP-1-102, that led to regression of human breast and lung cancer xenografts [198]. In a recent study, the authors showed that a potent and selective small-molecule degrader of STAT3 could produce complete tumor regression [199]. The inhibitor pyrimethamine displays anti-cancer activity and immune stimulatory effects in mouse breast cancer models [200]. Additionally, some FDA-approved compounds, such as pyrimethamine and celecoxib, have also been identified as STAT3 inhibitors [197]. Interestingly, another FDA-approved drug atovaquone was identified as a STAT3 inhibitor and anticancer agent using a gene expression-based discovery platform [201]. Currently, there are 18 clinical trials testing STAT3 inhibitors as cancer treatments listed in the database (clinicaltrials.gov), with 8 of these trials now completed, three recruiting, and one not yet recruiting.

As a note of caution when considering combination approaches, the JAK-inhibitor ruxolitinib has been shown to impair DC cell function both in vitro and in vivo [202]. To overcome this issue, the same team has identified a different JAK inhibitor, pacritinib, that can effectively conserve DC function when compared to ruxolitinib [203].

#### Combination of OVs with modulators of IFNγ-JAK-STAT pathway

The effects of STAT inhibitors on OV are complex and appear to depend on both selected OV and type of tumor. One of the major mechanisms of resistance to VSV infection is the type I IFN response, leading to the development of IFNβ-armed VSV. This VSV-IFNβ virus leads to resistance of viral replication in normal cells with intact IFN signaling but allows viral replication in cancer cells with defective IFNβ signaling. However, some cancer cells have intact or partially intact IFN signaling pathways and can resist VSV-mediated therapy. To overcome this issue, the authors utilized the JAK/STAT inhibitor, ruxolitinib, in combination with VSV-IFNβ to see if inhibition of the signaling could enhance VSV-IFNβ therapy for lung cancer [204]. Combination of ruxolitinib and VSV-IFNβ therapy resulted in a trend toward improved survival of mice, suggesting that further evaluation is needed. In another study, Nguyen and colleagues found that mutations in the IFNγ-JAK-STAT pathway cause resistance to ICI immunotherapy can increase sensitivity to OV infection and treatment [116]. This study also supports JAK inhibitor-OV combination for treatment-naïve melanomas without IFN signaling defects.

#### p53 pathway and its roles in cancer, immune function, and cancer immunity

p53 is one of the most well-studied tumor suppressors. *TP53* is mutated in approximately 50% of all human cancers. Interestingly, in the other ~ 50% of cancer carrying wild type p53, the signaling pathway is often disrupted at other interaction points [205]. The activity of p53 can be inhibited through the function of the negative regulators MDM2 and MDMX [206]. As the functional domains of the p53, mutation hot spots, and loss/gain of function mutations are out of the scope of this article, readers are referred to two excellent reviews for these details [207, 208].

As the p53 pathway is one of the most crucial signaling pathways in cells, it has been widely investigated as a target for developing anti-cancer drugs [205, 206, 209]. A series of small molecules targeting MDM2 and MDMX have been developed and studied in preclinical models and in clinical studies [209, 210]. Lane and colleagues have found that an old antibiotic, actinomycin D (ActD), when used at low dose, could mimic nutlin-3 and impart highly specific activation of p53 dependent transcription, and induce a reversible protective growth arrest in normal cells and enhance the activity of chemotherapy-induced killing of p53-positive human tumor cells [211]. At low cytostatic concentrations, ActD promotes ribosomal stress, which decreases MDM2 activity, resulting in p53 stabilization and activation. Chen et al. showed that ActD-induced p53 expression is mediated by AKT [212]. This property of ActD led to a series of clinical studies, including 6 phase III clinical trials using ActD in combination with other anti-cancer agents in a number of tumor indications [209]. Most of these trials are ongoing and final reports have not yet been published.

p53 is also functionally important in normal immune processes and antitumor immunity [213]. The key conclusion from recent studies is that wildtype p53 has fundamental roles in cancer immunity, however, mutations in p53 not only cripple wildtype p53 immune functions but also subvert the immune functions through its gain-of-functions [213]. The mutant p53 is associated with inflammation and immune dysfunction, indicating that it modulates immunity associated with cancer. In one key study, the authors identified a role for cancer-cell-intrinsic p53 as a key regulator of pro-metastatic neutrophils by using a panel of 16 distinct genetically engineered murine models of breast cancer. Mechanistic studies revealed that loss of p53 in cancer cells induced the secretion of WNT ligands that could stimulate tumor-associated macrophages to produce IL-1β, thus driving neutrophil infiltration and systemic inflammation [214]. In another study, the authors discovered a novel mechanism of targeting p53 for cancer immunotherapy. Activation of p53 in immature myeloid precursor cells was observed to dictate their differentiation into Ly6c^+^CD103^+^ monocytic antigen-presenting cells in tumors [215]. Increasing p53 expression using a p53-agonist drug elicits a sustained increase in Ly6c^+^CD103^+^ cells in tumors during immunotherapy, leading to markedly enhanced efficacy and duration of response [215].

#### Combination of OV with small molecules targeting p53-pathways

At this time, no combination of OV and a small molecule modulator of p53 has been assessed experimentally, based on our extensive literature search. However, Tagawa et al. studied how an MDM2 inhibitor could interact with an OV to produce synergistic cytotoxicity of cancer cells. Specifically, they found that an MDM2 inhibitor achieves synergized effect with an oncolytic AdV (Ad-delE1B) lacking E1B-55 kDa gene to target mesothelioma with wildtype p53 through augmenting NFI expression [216].

#### Epigenetic reprogramming in cancer cells and immune cells

DNA methylation, histone modifications including acetylation, methylation and phosphorylation, are major forms of epigenetic modifications that play vital roles in gene regulation, key biological processes and in cancer biology [217]. Under the influence of the TME and during activation, immune cells undergo epigenetic reprogramming to adapt to their environments and which directly impact the resulting cell differentiation and functionality [218, 219]. Therefore, small molecule inhibitors of enzymes involved in DNA methylation and histone modifications, can be explored to potentially reverse the suppressed functions of immune cells in the TME [28, 220, 221]. For example, epigenetic silencing of CXCL9/10 expression by promoter DNA methylation and H3K27me3 in tumor cells limits the infiltration of cytotoxic T cells, preventing tumor attack and promoting tumor growth [222, 223]. To reverse these effects, inhibitors of epigenetic enzymes DNMT or EZH2, alone or in combination, restore CD8^+^ T cell infiltration and improve sensitivity to ICI [223, 224]. We and others have showed that DNA demethylating agents can enhance cancer immunogenicity and induce de novo expression of cancer-testis antigens that improve responses to adoptive T cell therapy [225, 226]. These and other studies strongly suggested that epigenetic modulation may turn cold tumors hot [227, 228], thereby increasing the responsiveness of tumors to immunotherapy.

Another subtype of epigenetic modification is on SUMOylation. The covalent conjugation of small ubiquitin-like modifier (SUMO) proteins to protein substrates may lead to suppress type I interferon responses. Lightcap and colleagues recently studied the effects of TAK-981, a small-molecule SUMOylation inhibitor, on human and mouse immune cells. They found that the compound promoted the activation of dendritic cells and T cells. Further, they demonstrated that this compound activates antitumor immune responses and potentiates immune therapies in preclinical tumor models [229].

So far 10 small-molecule epigenetic drugs have been approved to treat several types of cancer (Table 2). Many other small molecule inhibitors targeting key enzymes involved in epigenetic pathways have been discovered and are at preclinical and clinical development as cancer therapies [230–232]. These emerging agents have the potential to be also explored in the context of immunotherapy, as has been recently reviewed [27, 28, 233].

**Table 2:** Approved small-molecule inhibitors of epigenetic enzymes for cancer treatments

Name	Target^a^	Cancer types	Approval time
Azacitabine	DNMT	Acute myeloid leukemia (AML); Chronic myelomonocytic leukemia (CMML); Myelodysplastic syndromes (MS)	2004
Decitabine	DNMT	AML; CMML; MS	2006
Vorinostat	HDAC	Cutaneous manifestations of cutaneous T-cell lymphoma (CTCL)	2006
Romidepsin	HDAC	CTCL	2009
Belinostat	HDAC	Relapsed or refractory peripheral T-cell lymphoma (PTCL)	2014
Panobinostat	HDAC	Multiple myeloma (MM)	2015
Chidamide	HDAC	Relapsed/refractory PTCL	2015
Enasidenib	IDH2	Relapsed or refractory AML	2017
Ivosidenib	IDH1	Relapsed or refractory AML	2018
Tazemetostat	EZH2	Epithelioid sarcoma and relapsed or refractory follicular lymphoma	2020

^a^*Abbreviations are*: *DNMT* DNA methyltransferase, *HDAC* histone deacetylase, *IDH1* and *IDH2* isocitrate dehydrogenase 1 and 2, *EZH2* enhancer of zeste homolog 2

#### Combination of OVs with inhibitors of epigenetic enzymes

One major hurdle in cancer therapy is heterogeneity of cancer, which results in variations in the ability of tumors to support productive infection by OVs and to induce adaptive anti-tumor immunity. Mounting evidence suggests tumor epigenetics may play a key role in this heterogeneity. In the last decade, therapeutic strategies aiming to exploit the epigenetic identity of tumors have been developed [28, 234, 235]. Some recent studies combining OV with epigenetic drugs are shown in Table 3.

**Table 3:** Preclinical studies of OV combining with small molecule epigenetic drugs

OV	Epigenetic Drug	Tumor type	Effects	References (1st author, year)
AdV: H101	TSA	Esophageal squamous cell carcinoma	1). Enhance viral replication and spread. 2). Improved antitumor activity	Ma, 2017 [236]
Herpes virus: GM-CSF-HSV	VPA (valproic acid)	(In vitro)	1). VPA enhances viral replication and GM-CSF production and oncolysis; 2). VPA improves antitumor immunity.	Jennings, 2019 [237]
BHV-1	Trichostatin A (TSA)	Lung cancer	1). TSA promotes viral replication; 2). TSA exacerbates DNA damage and cytopathology, suggesting a synergy between BHV-1 and TSA	Qiu, 2021 [238]
Rhabdoviridae: VSVΔM51	Vorinostat; MS275; SIRTi	Prostate cancer	1). SIRT1 inhibition promotes the permissivity of prostate cancer PC-3 cells to VSVΔM51 replication and oncolysis; 2). HDACi upregulated the microRNA miR-34a that regulated the level of SIRT1.	Muscolini, 2019 [239]
Reoviridae (RV): Reolysin	Entinostat Vorinostat	1). Squamous cell carcinoma. 2). Multiple myeloma	1). HDAC inhibition increased JAM-1 and reovirus entry, and viral replication; 2). The combination results in synergistic killing via apoptosis; 3). The combination improved immune cell infiltration and higher therapeutic efficacy.	Jaime-Ramirez, 2017 [240] Stiff, 2016 [241]
Reolysin	belinostat	Lymphoma	1). Belinostat-resistant lymphoma cell exhibit downregulated IRF1 and STAT1 expression; 2). These cells are hypersensitive to RV replication and induced cell death; 3). The combination therapy displays synergy.	Islam, 2020 [242]
Paramyxoviridae: MeV	Resminostat	Pancreatic cancer	1). Synergistic mode of cytotoxicity. 2). The HDACi neither impaired MeV growth kinetics nor prevented the activation of the interferon signaling pathway.	Ellerhoff, 2016 [243]
Parvoviridae: P/V-CP	Scripaid	Small cell lung cancer cells/laryngeal carcinoma cells	1). enhanced spread of the virus and cell apoptosis; 2). suppressed interferon-beta induction through blocking phosphorylation and nuclear translocation of IRF-3.	Fox, 2019 [244]

The table lists studies published in or after 2016 only

Histone deacetylases (HDACs) are a large family of enzymes that have crucial roles in numerous biological processes, largely through their repressive influence on transcription [245]. HDACi have been shown to inhibit the IFN response [246]. HDACs may modulate epigenetic modifications of histones and chromatin, and other cellular regulatory proteins (such as p53, E2F1–3), leading to diminished cellular antiviral response. In one study, two HDACi markedly enhance the infection and spread of VSV and OVV in primary human tumor tissue explants and multiple tumor models. In another study, two HDACi (Scriptaid and LBH589) combined with OV Delta24-RGD led to enhanced efficacy in patient-derived glioblastoma cells [247]. HDAC6 inhibition enhances oHSV replication in glioma [248]. This was in line with previous studies demonstrating that HDAC6 controls innate immune and autophagic responses to TLR-mediated signaling by intracellular bacteria [249], and thus likely viruses too.

Reduced cellular IFN responses and enhanced virus-induced apoptosis may explain the increased viral replication and oncolytic activity in some cases [250]. In one study, an unexpected property of HDACi on adaptive immunity was found [251]. Entinostat (MS-275), which is a class I-specific HDACi, induced lymphopenia, leading to selective depletion of bystander lymphocytes and Treg cells but allowed expansion of antigen-specific secondary responses. Intriguingly, during the boosting phase, coadministration of an oVSV with entinostat biased the immune response towards anti-tumor immunity by suppressing the primary anti-viral immune response and enhancing the boost response against tumor antigens. Overall, this HDACi enhanced OV-mediated therapeutic efficacy, suppressed autoimmunity and thus improved the therapeutic index [251].

In a recent study, Muscolini et al reported that the NAD^+^-dependent histone deacetylase SIRT1 plays a key role in the permissivity of PC-3 prostate cancer cells to VSVΔM51 viral replication and oncolysis. Enhanced VSVΔM51 infection and cancer cell killing were mediated by HDACs and directly correlated with a decrease of SIRT1 expression [239]. In addition, pharmacological inhibition sensitized prostate cancer cells to VSVΔM51 infection, resulting in augmentation of virus replication and spread. Mechanistically, HDACi upregulated the microRNA miR-34a that down-regulated the level of SIRT1 [239].

#### Immune checkpoint pathways

The discovery of key immune checkpoint molecules (CTLA-4 and PD-1) and their blockade as novel approaches in cancer immunotherapy led to the Nobel Prize in Medicine in 2018 [252]. Monoclonal antibodies against PD-1 or PD-L1 have been approved to treat various types of cancer with proven efficacy [253]. However, therapeutic antibodies face functional and practical limitations, including inadequate tissue accessibility, pharmacokinetic challenges, impaired interactions with the immune system, as well as high production costs [254]. The toxicities observed following delivery of anti-PD-1/PD-L1 antibodies are well documented [255]. The general adverse events (AEs) may include fatigue, pyrexia, infusion reactions; while organ-specific AEs may include dermatologic toxicities, diarrhea/colitis, endocrine toxicities, hepatic toxicities, and pneumonitis.

Small molecule inhibitors targeting PD-1/PD-L1 signaling pathway may provide an attractive alternative approach. The rational design strategies of these small molecules are based on three approaches as summarized in an excellent review paper by Wu et al. [256]: 1). Blocking direct interaction of PD-1 and PD-L1 proteins; 2). Inhibiting the expression (transcription and translation) of PD-L1 protein; 3). Promoting the degradation of the PD-L1 protein. Bristol-Myers Squibb (BMS) and Aurigene Discovery Technologies each disclosed several promising PD-1/PD-L1 inhibitors, including small molecules and peptides, although only a fraction of experimental data have been disclosed to the public [257, 258]. Following on this approach, other inhibitors have been discovered by both industry and academic groups. One recent study detailed a series of novel inhibitors for PD-1/PD-L1 interactions, with A9 as a standout [259]. We have listed some representative inhibitors in Table 4.

**Table 4:** Examples of small molecule modulators for immune checkpoints

*Name*	*Target*	*Research stage*	*Cancer types*	References (First author, year)
APBC A9 II-14 (d)PPA-1	PD-1 binding to PD-L1	Preclinical	Melanoma (in vitro) Colon cancer Colon cancer	Wang 2021 [260] Zhang 2021 [259] Fang 2021 [261] Chang 2015 [262]
JQ-1 (BRD4i) eFT508	The expression of PD-L1	Phase I Phase I	Lymphoma Liver cancer	Zhu 2016 [263] Xu 2019 [264]
PD-LYSO Curcumin Metformin	The degradation of PD-L1	Preclinical Preclinical Preclinical	(in vitro) Breast cancer Breast cancer	Wang 2019 [265] Lim 2016 [266] Cha 2018 [267]
CA-170	VISTA and PD-1/PD-L1 inhibitor	Phase I	Advanced tumors and lymphomas	Sasikumar 2021 [268]
NDI-101150	MAP4K1 (HPK1) inhibitor	Phase I/II	Solid Tumors	You 2021 [269]
NX-1607	CBL-B inhibitor	Phase I	Advanced malignancies	Loeser 2007 [270]
TNO155	SHP2 inhibitor	Phase I/II	Advanced Solid Tumors	LaMarche 2020 [271]

Basic and preclinical studies have been performed to assess the functional properties and potency of small molecules or peptides that interact with the PD-1/PD-L1 axis and how these agents impact antitumor immunity. In one study, the authors conducted systematic in vitro characterization on BMS-103, BMS-142 and others [272]. These compounds are strongly active in biochemical assays, yet their acute cytotoxicity greatly compromised their immunological activity. In contrast, Wang et al. have found that a new small molecule inhibitor, called APBC, could effectively interrupt the PD-1/PD-L1 by directly binding to PD-L1, presenting the K_D_ and IC_50_ values at low-micromolar levels. Better yet, it could elevate cytokine secretions of the primary T-lymphocytes that are cocultured with cancer cells in a dose-dependent manner. It displayed superior antitumor efficacy in B16F10 melanoma in hPD-L1 knock-in mouse model without the induction of observable liver toxicity [260]. Koniecny and the team have developed di-bromo-based small molecule inhibitors for PD-1/PD-L1 interaction and showed its activity in vitro [273]. Fang et al. discovered 1,3,4-oxadiazole derivatives as inhibitors for PD-1/PD-L1 interaction and found compound II-14 as the most potent one in biochemical activity and antitumor activity [261].

It is important to note that small molecules have been developed for targeting additional immune checkpoints. Cbl-b is expressed in all leukocyte subsets and regulates multiple signaling pathways in T cells, NK cells, B cells, and some types of myeloid cells. Cbl-b negatively regulates activation signals through antigen or pattern recognition receptors and co-stimulatory molecules. Several studies showed that Cbl-b-gene knockout mice reject tumors. Thus, targeting Cbl-b may be an promising strategy to enhance antitumor immunity [274]. Src homology-2-containing protein tyrosine phosphatase 2 (SHP2) is a major phosphatase involved in several cellular processes. Recent studies showed that this enzyme plays vital roles not only in T lymphocytes and macrophages, but also cancer cells. Thus, exploring the use of SHP2 inhibitors has potential promise for cancer immunotherapy [275]. Hematopoietic progenitor kinase 1 (HPK1/MAP4K1) is a hematopoietic cell-restricted member of the serine/threonine Ste20-related protein kinases. It is a negative regulator of the T cell receptor, B cell receptor, and DCs. It is probable that blocking the HPK1 kinase activity with a small molecule inhibitor may elicit superior anti-tumor activity of both T and B cells, resulting in a synergistic amplification of anti-tumor potential [269, 276]. Small molecule inhibitors for these enzymes have been developed and some of them are in clinical studies for cancer immunotherapy (Table 4).

#### Combination of OV with immune checkpoint blockade

As we have noticed, the discovery of small molecule modulators for PD-1/PD-L1 (Table 3) are relatively recent events, thus no studies combining them with OVs have been published. However, many studies with OVs in combination with immune checkpoint blockade using PD-1, PD-L1, CTLA-4 antibodies have studied. Many reviews on this topic have been published and we refer to two recent great reviews [7, 277]. Worth specific mentioning are two clinical studies of T-VEC with either anti-PD-1 or anti-CTLA-4 antibodies, achieving dramatically improved clinical responses with up to ~ 67% objective response rate in patients with advanced melanoma [55, 105].

#### Other pathways of particular importance to innate immunity

The cGAS–STING signaling pathway is a key mediator of inflammation and innate immunity in settings of infection, cellular stress, and cancer [278]. A network of pattern recognition receptors (PRRs) can detect invading viruses and trigger the host antiviral response. Stimulator of interferon genes (STING) functions as a node and integrator of the signal network—now called cGAS-STING-TBK1 pathway [279]. At this time, the cGAS-STING-TBK1 axis is considered the major signaling pathway in innate immune response across different species. Indeed, cGAS-STING is an important pathway in cancer immunotherapy [280]. In addition to their role in antitumor immunity, STING agonists have recently been shown to be effective in promoting tumor vascular normalization and formation of tertiary lymphoid structures within the therapeutic TME [281].

Small molecules targeting this innate immune signaling pathway have been developed and some are now undergoing clinical testing [282]. It is noteworthy that high potency STING agonists can engage unique myeloid pathways to reverse pancreatic cancer immune privilege [283]. In this study, potent synthetic STING agonists (e.g., IACS-8803) reprogrammed suppressive myeloid populations, both human and murine origins, in part through inhibition of Myc signaling, metabolic modulation, and antagonism of the cell cycle. Inhibitors of upstream molecules may also lead to the activation of STING pathway. CX-5461 is one of the most promising inhibitors for RNA polymerase I, and previous reports have shown that CX-5461 treatment induces DNA damage response through ATM/ATR kinase. In a recent study, the authors reported that CX-5461 could induce a rapid accumulation of cytosolic DNA. This accumulation led to transcriptional upregulation of STING, phosphorylation of IRF3, and activation of type I interferon response. Thus, CX-5461 therapy-induced immune activation may be exploited as novel drug combinations with the potential to increase immunotherapy efficacy [284]. In another study, inhibition of Ataxia telangiectasia mutated (ATM) enhanced cancer immunotherapy by promoting mitochondrial DNA leakage and cGAS/STING activation [285]. The authors suggested that that ATM may serve as both a therapeutic target and a biomarker to enable ICI therapy.

#### Combination of OVs with small molecule modulators of other pathways

The STING axis may be silenced during malignant transformation, allowing cancers to escape immune surveillance, which may in turn allow OV to efficiently replicate and exert therapeutic benefit in these cancers [286]. A recent study demonstrated that STING restricts oHSV replication and spread in resistant malignant peripheral nerve sheath tumors [287]. However, while STING knockout tumors could support increased lytic potential by HER2-retargeted oHSV-1, these tumors also showed molecular signatures of an immunosuppressive TME. These signatures were correspondingly associated with ineffectiveness of the combination therapy in a tumor model. Accordingly, these authors proposed that antiviral, tumor-resident Sting provides a fundamental contribution to immunotherapeutic efficacy of OV [288]. Kaufman, Rabkin and associates conducted a study that suggests T-VEC induces ICD and promotes tumor immunity, and it can induce therapeutic responses in anti-PD-1-refractory, low STING-expressing melanoma [155]. To summarize, these data that OV immunotherapy induces ICD and overcomes STING deficiency in melanoma.

Some studies have explored inhibitors targeting the transforming growth factor beta receptor 1 (TGF-βR1). Cripe and associates combined OV HSV1716 with an TGF-βR1 inhibitor (A8301) to treat syngeneic models of murine rhabdomyosarcoma [289]. They observed enhanced efficacy appears to depend on an improved anti-tumor T cell response. In another study with patient-derived recurrent GBM models, dual therapy with an oHSV and inhibitors of TGF-β receptor kinase also led to enhanced efficacy [290]. These studies revealed a novel synergy of virotherapy and blockade of TGF-β signaling and warrant further preclinical investigation to support clinical translation of this combination strategy. In addition, these two agents, either alone or in combination, can increase the susceptibility of immune-silent tumors to immune checkpoint therapy [291].

Aurora-A kinase is also a promising therapeutic target in cancer [292]. Two studies showed that, the kinase inhibitors alone or in combination with OVs, can possess additive/synergistic killing effects on cancer cells [293–295]. Interestingly, in one study, the authors found that aurora A kinase inhibition enhances oHSV virotherapy through cytotoxic synergy and innate cellular immune modulation, noting that alisertib inhibited virus-induced accumulation of intra-tumoral MDSCs [295].

Small molecule experimental drugs can target cancers with intrinsic or acquired resistance to the infection of various OVs. Xiao et al. have recently demonstrated that DNA-dependent protein kinase (DNA-PK) inhibition sensitizes cancer cells to alphavirus M1 and improves therapeutic effects in refractory cancer models and in patient tumor samples. It turned out that DNA-PK inhibition synergizes with OV M1 by inhibiting antiviral response and potentiating DNA damage [296].

One mechanism of OV therapeutic failure is tumor cell resistance to OV infection [297]. Dimethyl fumarate (DMF) is a common treatment for psoriasis and multiple sclerosis, and it also exhibits anticancer properties. Selman et al. observed that DMF and various fumaric and maleic acid esters (FMAEs) could enhance viral infection of cancer cells as well as human tumor biopsies by OVs, including VSV, AdV and HSV. Together, these combination therapies improved therapeutic outcome in OV-resistant syngeneic tumors and xenograft models in mice [298]. This study demonstrates that unconventional application of FDA–approved drugs and biological agents can result in improved anticancer therapy.

ICD inducers are useful in induction of cancer cell death and eliciting antitumor immunity [299]. Most OVs themselves are ICD inducers [30]. However, small molecules can also function as ICD inducers, further potentiating the induction of antitumor immunity when combined with OVs. For example, CDK4/6 inhibitor and VSVΔ51 synergistically induced ICD and boosted antitumor immunity, leading to enhanced efficacy in refractory glioblastoma [300]. Mechanistically, CDK4/6 inhibition led to autophagic degradation of MAV, resulting in dampened antiviral responses and thus enhanced tumor-selective replication of the OV. This CDK4/6 inhibition cooperated with OV infection to induce severe DNA damage stress and amplify ICD, leading to increased numbers of activated CD8^+^ cells [300]. Another study tested the combination of a ferroptosis enhancer with an OV. Erastin itself is a typical activator of ferroptosis, considered to be one type of ICD. We showed that Erastin alone had a limited effect on systemic immunity or localized intratumoral immunity in tumor-bearing mice. When combined with an OV, however, erastin enhanced the number of activated DCs and the activity of tumor-infiltrating T lymphocytes (based on increased IFN-γ^+^CD8^+^ and PD-1^+^CD8^+^ T cells), leading to improved therapy in tumor models in mice [301].

Efforts have been undertaken to identify novel small molecules that can work with OVs for improved antitumor effects. Diallo et al. uncovered a novel small molecule to target the complex cellular defense mechanisms that permit effective viral infection and replication. They have designed a high-throughput pharmacoviral approach and this identified novel chemical sensitizers (e.g., VSe1) to allow effective infection and replication of OVs such as VSVΔ51 [302]. Through this screen, they identified compound #1 that can sensitize resistant cancer cells to infection with VSVΔ51 by damping the activation of antiviral responses in cancer cells. However, compound 1 is rapidly degraded and thus derivatives with improved stability and activity would be desirable. To achieve this aim, Dornan et al endeavored to develop lead compounds (pyrrole-derivatives) that increased OV growth up to 2000-fold in vitro and demonstrated remarkable tumor selectivity both ex vivo and in vivo [303]. This expands the scope of OVs to include the originally resistant tumors, further potentiating this promising therapy. In another study, investigators found that vanadium compounds can potentiate oncolytic immunotherapy though multiple mechanisms [304]. The compounds work by subverting the antiviral type I IFN response toward a death-inducing and pro-inflammatory type II IFN response, leading to improved OV spread, increased bystander killing of cancer cells, and enhanced antitumor immunity [304].

Combinations of OV with small-molecule modulator-containing cocktails is a highly effective strategy in modulating the TME and enhancing therapeutic efficacy. For example, Kalinski and colleagues demonstrated that IFN-α and polyinosinic: polycytidylic acid (p-I:C) synergize with the ‘classical’ type-1-polarizing cytokine cocktail (TNFα/IL-1β), allowing for serum-free generation of fully mature type-1-polarized DCs [305]. Further studies indicated that such polarized DCs produce much higher levels of IL-12p70 and induces up to a 40-fold increase in long-lived CTLs specific for melanoma-associated antigens. Later, the slightly modified triple cocktail (IFN-α, poly I:C, and a COX-2 inhibitor), termed chemokine modulating cocktail (CKM), was used for a series of preclinical and clinical studies [306]. The sequential treatment with an OV followed by CKM resulted in the upregulation of Th1-attracting cytokines (CKs) and reduction of Treg-attracting CKs (CCL22 and CXCL12), concurrent with enhanced trafficking of tumor-specific CD8^+^ T cells and NK cells into the TME. As a result, we observed highly significant antitumor activity and long-term survival of tumor-bearing mice [58]. Additionally, rapamycin and celecoxib have been evaluated in combination with OV and adoptive T cell transfer (ACT) [307]. The authors hypothesized that the combination of local tumor-specific T cells activation delivered alongside an anti-immunosuppressant would improve therapy for gliomas. They utilized an IL15Rα-IL15-encoding OV as the T-cell activating stimulus and a prostaglandin synthesis inhibitor as the anti-immunosuppressant, together with ACT of tumor-specific T cells. IL15Rα-IL15-armed OVs, in conjunction with ACT, rapamycin, and celecoxib provide potent antitumor effects against brain tumors, suggesting that complex but rationally designed therapeutic combinations can produce robust anti-tumor effects.

### OV in combination with other regimens

Conventional approaches to cancer therapy, such as chemotherapy, radiotherapy, and other types of immunotherapy have been well studied and combining OV with these classes of antitumor agents have been widely investigated. As these are outside the scope of this review, we will only briefly discuss these strategies. Readers are referred to recent reviews that have included more intensive discussion [6, 7, 9, 308].

Some traditional chemotherapeutic and targeted agents are now known to function, at least in part, through immunologic mechanisms [309]. If a particular chemo- agent and a particular OV work to target different immune cells and/or mechanisms that are favorable for antitumor immunity, it is likely that they may act synergistically to potentiate antitumor immunity and therapeutic efficacy.

In addition to surgery, radiotherapy remains the preferred treatment for locoregional tumors. Radiation therapy and chemotherapy are designed to target cancer cells by compromising cellular integrity during cell division. However, these agents can also induce immune modulation that can either impede or augment overall therapeutic efficacy. The impact of radiotherapy, as well as chemotherapy, on the immune system depends highly on context, making it challenging but imperative to understand how each cytotoxic therapy may compromise immune function [310].

It is interesting to note that radiotherapy has been used as a tool to elicit changes in clinically actionable signaling pathways in cancer [311] which can serve as targets for small molecule inhibitors in combination therapy. In this regard, radiotherapy and OV combinations have been evaluated, with some important preclinical observations that may support further study. Importantly, some armed OVs can replicate and express genes of interest selectively in tumor cells, thus improving noninvasive precision molecular imaging and radiotherapy [312]. Oncolytic VVs in combination with radiation have been extensively studied in tumor models [313–316]. Stereotactic body radiation combined with oncolytic VV induces potent anti-tumor effects by triggering tumor cell necroptosis and DAMPs, markers of ICD [317].

In another study, Delta-24-RGD has been combined with radiotherapy in diffuse intrinsic pontine glioma and pediatric high grade glioma models [318]. The combination led to synergistic anti-glioma effects. Interestingly, OV treatment led to the downregulation of relevant DNA damage repair proteins, further sensitizing tumors cells to radiotherapy. This is a rational combination and serves as a guide for clinical studies. Considering the clinical context and potential optimism for this combination approach, a recent clinical trial with oncolytic DNX-2401 followed by radiotherapy in pediatric patients with diffuse intrinsic pontine glioma [319] produced early promising outcomes. A total of 12 patients received up to 1 × 10^10^ or 5 × 10^10^ VP of DNX-2401 and 11 received subsequent radiotherapy. Intra-tumoral infusion of OV followed by radiotherapy resulted in changes in T-cell activity and a reduction in or stabilization of tumor size in some patients but was associated with adverse events.

## Clinical studies of combination strategy

We have conducted an extensive survey of the literature and ongoing clinical trials (clinicaltrials.gov). Only a few current clinical trials meet the specified criteria and involve delivery of OV with small molecules targeting key signaling pathways. These trials are listed in Table 5. From these analyses, five types of combinations were identified: OV combined with RTKi (sorafenib or apatinib), non-receptor TKI (Ruxolitinib, an inhibitor to Janus kinase, or JAK), irinotecan (topoisomerase inhibitor), or cyclophosphamide (immunomodulatory agent), or traditional chemotherapeutic agents (Carfilzomib; Gemcitabine; Nab-paclitaxel).

**Table 5:** Clinical trials for combination of OV with small-molecule modulators^a^

Identifier	OV	Modulator	Disease setting	Status
NCT02705196	LOAd703 (AdV)	Gemcitabine Nab-paclitaxel	Pancreatic cancer	Phase I/II/Recruiting
NCT05113290	AdV	Sorafenib	HCC	Phase IV/Active, not recruiting
NCT05070221	rHSV2hGM-CSF (HSV)	Axitinib (RTKi) + anti-PD-1 mAb	Melanoma stage IV	Phase I/Not yet recruiting
NCT03152318	rQNestin34.5v.2 (HSV)	Cyclophosphamide	Recurrent malignant glioma	Phase I/Recruiting
NCT03866525	OH2 (HSV)	+/− irinotecan	Solid tumor/GI cancer	Phase I/II/Recruiting
NCT02562755	Pexa-Vec (VV)	Sorafenib (RTKi)	HCC	Phase III/Completed
NCT03605719	Pelareorep (Reovirus)	Carfilzomib	Recurrent plasma cell myeloma	Phase I/Active, not recruiting
NCT03017820	VSV-hIFN-NIS (VSV)	Ruxolitinib (JTKi)^b^ (+/− Cyclophosphamide)	Multiple myeloma, Acute myeloid leukimia, T-cell lymphoma	Phase I/Recruiting
NCT03120624	VSV-hIFN-NIS (VSV)	Ruxolitinib (JTKi)	Endometrial cancer	Phase I/Recruiting
NCT04665362	M1-c6v1 (M1)	*Apatinib (RTKi)* Anti-PD-1 mAb	Advanced/metastatic HCC	Phase I/Not yet recruiting
NCT01394939	Pexa-Vec (VV)	+/− Irinotecan (Topoisomerase I inhibitor)	Metastatic, refractory colorectal carcinoma	Phase I/II /Completed
NCT00450814	MV-NIS (MV)	Cyclophosphamide	Recurrent or refractory multiple myeloma	Phase I/II /Completed

^a^ The clinicaltrials.gov database was searched using key words of ‘oncolytic virus’ and ‘inhibitor’ and a total of 28 studies were found (updated 8/29/2022). Some alternative key words were also used to find other clinical studies. Upon screening, only those meeting the criteria of “combination of OV with small molecule modulator” are listed

^b^ *JTKi* Janus (tyrosine) kinase inhibitor

It is worth emphasizing that ICI has now become standard of care for a variety of cancers. Many preclinical studies evaluating the combination of OVs with anti-PD-1/PD-L1 or anti-CTLA4 monoclonal antibodies have been performed with impressive therapeutic efficacy. Two clinical studies on melanoma patients, using T-VEC and either anti-PD-1 or anti-CTLA-4 antibody, have demonstrated striking efficacy for this combinatorial approach [55, 105]. However, as previously noted a phase III trial with anti-PD-1 and T-VEC failed to achieve better clinical response than anti-PD-1 alone in melanoma [96]. So far, small molecule inhibitors for checkpoint molecules PD-L1, MAP4K1, CBL-B and SHP2 are being investigated in clinical studies (Table 3). As such, the combination of an OV with small molecule ICI should be initiated within next few years.

While clinical trials combining OV with small molecule inhibitors are currently ongoing, the definitive data on their toxicity still await publication. Previous trials using OV or small molecule inhibitors as single agents generally indicate limited OV toxicity while small molecule inhibitors tend to induce more toxicities. For this reason, we expect toxicities resulting from these combination regimens to be mostly driven by the small molecule component, although some toxicities may expect to be potentiated by OV-driven effects. Therefore, successful combination therapies may require careful consideration and selection of small molecule inhibitor(s) with known activity/efficacy and limited toxicity as important criteria.

## Conclusions and perspectives

The clinical success of anti-CTLA4 and anti-PD-1/PD-L1 ICIs has led to vast expansion of the immuno-oncology field and the combination approaches to cancer therapy currently under investigation. Among them, the use of OV is an exciting and emerging branch, with three OVs showing clear efficacy in three cancer indications that led to subsequent approval in three countries. OVs work through multiple mechanisms, and act locally but function systemically via adaptive antitumor immunity. On the other hand, the development of small molecules targeting key signaling pathways continues to play an expanding role in immuno-oncology, however in most cases their efficacy as monotherapies has been limited. Logically, these two classes of antitumor agents can be combined, likely by using mechanistic insights to guide rational approaches to further improve treatment efficacy. Preclinical studies have demonstrated that combining OVs with small molecule modulators of key signaling pathways in cancer- and/or immune cells can have enhanced therapeutic efficacy (Figs. 2 and 3). Ongoing and upcoming clinical studies of these rational combinations will ultimately determine their relevance for treating human cancer.

Multiple factors are likely to determine the efficacy of these combinations. First, oncogenic signaling pathway have emerged as key targets for small molecules. These signaling pathways (such as AKT-PI3K-mTOR, KRAS-ERK/MAPK) play important functional roles not only in tumor development and progression, but also essential roles in non-transformed healthy cells, including immune cells. In this regard, the potential interactions between small molecule inhibitors, cancer cells, and immune cells may be complex and, in some cases, result in opposing effects. Importantly, the inhibitor may not only inhibit or kill cancer cells, but also suppress functions of immune cells, resulting in antagonistic, rather than synergistic or additive action. For example, mTOR, a vital sensor of signals within the immune microenvironment, is a central regulator of T cell biology [320]. Thus, mTOR inhibition may lead to disastrous effects and abrogate antitumor immunity. Extreme caution needs to be exercised when employing such inhibitors as part of a combination regimen, with careful understanding of the mechanistic impacts considered. Second, as many of the targetable signaling pathways are essential in normal cells, the potential toxicity induced by these signaling inhibitors will need to be carefully evaluated, monitored, and/or mitigated. Third, how the activity of small molecules intersect with the biological effects of OV need to be carefully evaluated. Specifically, if a small molecule induces cancer cell death prior to productive infection/replication by OV that is sufficient to produce robust oncolysis, then these agents may again functionally antagonize and limit overall therapeutic efficacy. Fourth, inhibitors specific for a mutated signaling molecule in cancer cells, such as KRAS^G12C^, are ideal ones to be explored in combination regimens, as they may provide much higher specificity for tumor targeting and thus would be anticipated to limit toxicity. The recently approved small molecule drug, Sotorasib, a highly specific inhibitor for KRAS^G12C^ protein, is such an example [137]. Our unpublished study showed that the combination of a small molecule inhibitor of KRAS^G12C^ with an OV elicited potent antitumor immunity and led to regression of tumors with KRAS^G12C^-mutant protein (Zhu Z et al., submitted for publication). Lastly, depending on the mechanism of action driving potential synergy between agents, the timing and order of the treatments could have significant effects on treatment outcome [4] and may directly determine whether the combined effects are synergistic/additive, or potentially reverse any beneficial effects. For example, it has been shown that sequential administration with OV Pexa-Vec (JX-594), followed by Sorafenib, results in better efficacy in HCC [321]. In that study, a potential problem with this combination emerged where if given simultaneously, sorafenib could block Pexa-Vec replication through RAF inhibition. The study’s authors explored simultaneous and sequential combinations in vitro and in animal tumor models, finding that the regimen of Pexa-Vec followed by sorafenib was statistically superior to reverse approach. Importantly, additional well designed mechanistic studies are needed to identify optimal combinations, as the combination of two classes of agents may produce novel and/or unexpected mechanisms of action at the molecular, cellular, and/or host level.

Novel experimental systems and cutting-edge technologies that can be used to dissect complex mechanism(s) of action help to facilitate the development of novel combination regimens, inform clinical studies and, eventually, can help establish new standards of treatment or therapeutic paradigms These developments include but are not limited to: 1) Three-dimensional organoids that represent a promising, near-physiological model for human cancers and tremendously support diverse potential applications in cancer research including immunotherapy and OV combinations [322], and 2) human tumor tissue explant cultures provide patient-derived models for short term studies (data available within days, rather months) and involve all components of the TME, including cancer, stroma, inflammatory lymphoid and myeloid infiltrate, as well as vasculature (although without active blood flow) [306]. The results from these simplified in vitro systems may guide in-depth in vivo studies, saving time and costs. In addition, novel tools to analyze the immune landscape of tumor tissues before and after combination therapy may provide useful data to further inform combination strategies, with single cell RNA sequencing (scRNA Seq) and proteomics providing two powerful examples. In fact, scRNA Seq has been used to analyze the immune profile in OV-immunotherapy [323]. Finally, CRISPR/Cas9 knockout of selected candidate genes provides a breakthrough technology to study gene functions in cancer in vivo [324]. More importantly, in vivo CRISPR screens have identified important regulators of antitumor immunity and candidate targets for cancer immunotherapy [325, 326]. We envision that these powerful technologies and experimental systems will strongly accelerate the identification and translation of novel combination regimens into highly effective immunotherapies for cancer patients.

At this time, while clinical trials evaluating these approaches have been limited and have generally focused on OV in combination with RTKi and other TKi, topoisomerase inhibitor, or cyclophosphamide, new opportunities are rapidly emerging. As preclinical studies have shown that inhibitors of EGFR/KRAS/MAPK, HDACi and certain metabolic enzymes effectively synergize with OVs, these approaches are likely to translate into new therapies for clinical testing. In worst cases, even if some combinations may not deem to be rational, they may still work better than either monotherapy as one study explained the superiority of many combinations of approved drugs in the absence of drug synergy or additivity [37].

With increased understanding of cancer biology, and improved small molecule inhibitor/modulators available, it will be necessary to explore additional combinations of OV with modulators of signaling pathways in future preclinical studies to identify optimal approaches. As a branch of precision personalized medicine, one of the biggest challenges is to manage the most efficient ways to identify which combination to use, when to use them, and which patients are most likely to benefit from these approaches.

## Data Availability

The materials supporting the conclusions of this review has been included within the article.

## Abbreviations

AdV: Adenovirus
ATM: Ataxia telangiectasia mutated
BCG: Bacillus Calmette-Guérin vaccine
CAF: Carcinoma-associated fibroblasts
CK: Chemokine
CKM: Chemokine modulating cocktail
CTL: Cytotoxic T lymphocytes
DAMP: Damage-associated molecular pattern
DC: Dendritic cells
EGFR: Epidermal growth factor receptor
4E-BP1: Eukaryotic translation initiation factor 4E-binding protein 1
GBM: Glioblastoma
HCC: Hepatocellular carcinoma
HDAC: Histone deacetylase
HSV: Herpes simplex virus
ICD: Immunogenic cell death
ICI: Immune checkpoint inhibitor
JTKi: Janus (tyrosine) kinase inhibitor
MV: Measles virus
MDSC: Myeloid-derived suppressor cells
mTOR: The mammalian target of rapamycin
Myx: GTP-binding protein MX
NDV: Newcastle disease virus
NSCLC: Non-small cell lung cancer
OAS: Oligoadenylate synthetase
oHSV: Oncolytic HSV
OV: Oncolytic virus
PAMP: Pathogen-associated molecular pattern
Pexa-Vec: Pexastimogene devacirepvec
PD1: Programmed cell death 1
PD-L1: Programmed cell death ligand-1
PGE2: Prostaglandin E2
PI3K: Phosphoinositide 3-kinases
PKR: Protein kinase R
RCC: Renal cell carcinoma
ROS: Reactive oxygen species
RTK: Receptor tyrosine kinase
STING: Stimulator of interferon genes
TAM: tumor-associated macrophages
TGF-β: Transforming growth factor-β
TME: Tumor microenvironment
TKI: Tyrosine kinase inhibitor
T-VEC: Talimogene laherparepvec
VSV: Vesicular stomatitis virus
VV: Vaccinia virus
